# The stress-responsive kinase DYRK2 activates heat shock factor 1 promoting resistance to proteotoxic stress

**DOI:** 10.1038/s41418-020-00686-8

**Published:** 2020-12-02

**Authors:** Rita Moreno, Sourav Banerjee, Angus W. Jackson, Jean Quinn, Gregg Baillie, Jack E. Dixon, Albena T. Dinkova-Kostova, Joanne Edwards, Laureano de la Vega

**Affiliations:** 1 Division of Cellular Medicine, School of Medicine, University of Dundee, Dundee, Scotland UK; 2 Department of Pharmacology, University of California San Diego, La Jolla, CA 92093-0721 USA; 3 Unit of Gastrointestinal Oncology and Molecular Pathology, Institute of Cancer Sciences, College of Medical, Veterinary, and Life Sciences, University of Glasgow, Glasgow, UK; 4 Department of Cellular and Molecular Medicine, University of California San Diego, La Jolla, CA 92093 USA; 5 Department of Chemistry and Biochemistry, University of California San Diego, La Jolla, CA 92093 USA

**Keywords:** Cancer, Cell biology, Protein folding, Prognostic markers

## Abstract

To survive proteotoxic stress, cancer cells activate the proteotoxic-stress response pathway, which is controlled by the transcription factor heat shock factor 1 (HSF1). This pathway supports cancer initiation, cancer progression and chemoresistance and thus is an attractive therapeutic target. As developing inhibitors against transcriptional regulators, such as HSF1 is challenging, the identification and targeting of upstream regulators of HSF1 present a tractable alternative strategy. Here we demonstrate that in triple-negative breast cancer (TNBC) cells, the dual specificity tyrosine-regulated kinase 2 (DYRK2) phosphorylates HSF1, promoting its nuclear stability and transcriptional activity. DYRK2 depletion reduces HSF1 activity and sensitises TNBC cells to proteotoxic stress. Importantly, in tumours from TNBC patients, DYRK2 levels positively correlate with active HSF1 and associates with poor prognosis, suggesting that DYRK2 could be promoting TNBC. These findings identify DYRK2 as a key modulator of the HSF1 transcriptional programme and a potential therapeutic target.

## Introduction

Approximately 90% of solid tumours and 75% of hematopoietic cancers exhibit some degree of aneuploidy [1]. Evidence from yeast and mammalian systems has shown that aneuploid cells are less fit than their euploid counterparts [2, 3]. This is believed to be due to gene dosage imbalances, which cause the accumulation of excess and often misfolded proteins that must be chaperoned and/or degraded in order to prevent proteotoxic stress [4, 5]. To survive these aneuploidy-related imbalances, cells engage protein degradation and folding pathways [6, 7], and one might predict that positive regulators of these pathways are critical for the survival of aneuploid cells, and thus represent potential cancer therapeutic targets.

Overexpression of heat shock factor 1 (HSF1), the master regulator of the proteotoxic stress response, attenuates the negative effects of extra chromosomes on protein folding [6]. This agrees with the established critical role that HSF1 plays in tumour initiation [8] and in promoting and maintaining cancer cell proliferation [8, 9]. High levels of HSF1 expression have been reported in a number of cancers [10] and high levels of nuclear HSF1 are associated with poor outcome in breast, hepatocellular and endometrial carcinoma [11–13] among others. Under basal conditions, HSF1 is maintained largely in the cytoplasm and upon proteotoxic stress, HSF1 is activated and accumulates in the nucleus [14]. Once in the nucleus, HSF1 activates a comprehensive transcriptional programme including a number of genes encoding heat shock proteins (HSPs), which function as molecular chaperones and act to protect cells against proteotoxic stress and apoptosis [15]. Of the HSPs, *HSP70*, is one of the best-characterised HSF1 targets, and is expressed at high levels in a variety of cancers [16]. HSF1 activity and stability are tightly controlled by multiple posttranslational modifications [17]. Among these, phosphorylation of serine 320 and serine 326 is associated with enhanced transcriptional activity, stability and nuclear accumulation of HSF1 [18–20]. Importantly, the phosphorylation of S326 is both critical and dominant over any inhibitory phosphorylation events and is thus considered a hallmark of HSF1 activation [10].

Here we report that DYRK2 phosphorylates HSF1 increasing its nuclear stability and supporting its transcriptional activity, and thus promoting resistance to proteotoxic stress. We further show that in clinical TNBC samples DYRK2 protein levels correlate with active HSF1, and are associated with high rates of tumour recurrence and poorer patient survival. Taken together, our data suggest that DYRK2 is a hitherto unknown major regulator of the HSF1 pathway, which acts to promote TNBC cell survival. TNBC is a tumour type characterised by genomic instability and aneuploidy, high rate of recurrence and metastases, for which current therapeutic options are limited [21]. Inhibition of DYRK2 may thus represent a novel approach to compromise cellular proteostasis and increase the therapeutic options for TNBC.

## Results

### DYRK2 phosphorylates HSF1

**Fig. 1:**
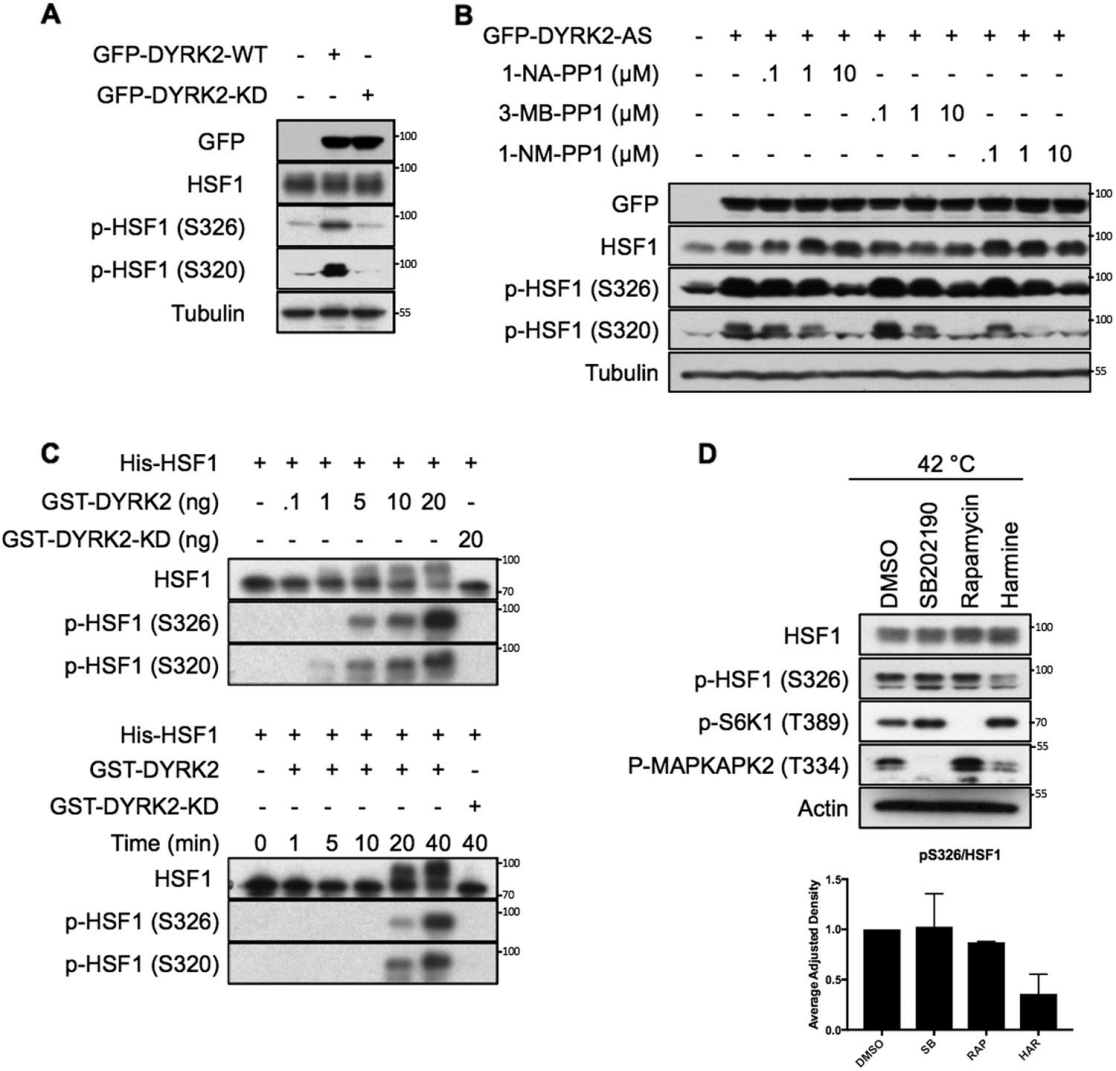
DYRK2 phosphorylates HSF1. **A** 293T cells were transiently transfected to express GFP-tagged DYRK2 wild-type (WT) or a kinase dead (KD) version. After 48 h, cells were lysed and the levels of endogenous HSF1 and phospho-HSF1 were analysed as indicated. **B** 293T cells were transiently transfected with a GFP-tagged DYRK2 analogue sensitive (AS) version. After 48 h, cells were treated for a further 3 h with increasing concentrations of three different PP1 inhibitors as indicated. Cells were lysed and the levels of endogenous HSF1 and phospho-HSF1 were analysed by western blot. **C** Upper panel, purified recombinant His-HSF1 (1 µg) was incubated in kinase buffer with increasing concentrations of recombinant GST-DYRK2 or GST-DYRK2 kinase dead (KD) at 30 °C for 30 min. Lower panel, purified recombinant His-HSF1 (1 µg) was incubated with either 20 ng of GST-DYRK2 WT or KD at 30 °C for various times as indicated. The reactions were terminated by the addition of SDS gel loading buffer, the proteins were resolved by SDS-PAGE, and the levels of phosphorylated HSF1 were analysed. **D** MDA-MB-468 cells were treated with either DMSO, the p38 inhibitor SB202190 (10 µM), the mTOR inhibitor rapamycin (30 nM) or harmine (10 µM). After 1 h, cells were incubated at 42 °C. After 1 h, cells were lysed in SDS buffer and the levels of the indicated proteins were analysed. Upper panel is a representative western blot and the bottom panel shows the quantification of the ratio between the phospho-HSF1 and total HSF1 levels. Data represent means ± SD (*n* = 3) and are expressed relative to the DMSO treated samples. See also Fig. S1.

The dual specificity protein kinase YAK1, a member of the DYRK family, phosphorylates and activates HSF1 in *Saccharomyces cerevisiae* [22]. To test whether in a similar way DYRK2 phosphorylates and activates HSF1 in human cancer cells, we overexpressed DYRK2 and, by use of phosphospecific antibodies, we observed that the levels of endogenous HSF1 phosphorylated at S326 and S320 (two main phosphorylation events linked to HSF1 activation) were increased (Fig. 1A). The kinase activity of DYRK2 was required for the increased levels of pS326- and pS320-HSF1, as a kinase-dead version of DYRK2 (DYRK2-KD) did not induce HSF1 phosphorylation.

To further test the role of DYRK2 kinase activity on HSF1 phosphorylation, we mutated the gatekeeper residue within DYRK2, creating an analogue-sensitive DYRK2 form (DYRK2-AS) that is selectively sensitive to PP1 inhibitors [23]. Using the DYRK2-AS form together with three different PP1 inhibitors, we observed that specific inhibition of DYRK2 activity, dose-dependently reduced the DYRK2-mediated phosphorylation of endogenous HSF1 (Fig. 1B). In addition, harmine, a well-characterised pan-specific DYRK inhibitor [24] (Fig. S1A) that inhibits DYRK2 at concentrations in the µM range [25–28] also reduced the phosphorylation of endogenous HSF1 mediated by DYRK2 overexpression (Fig. S1B).

To address if HSF1 is a direct substrate for DYRK2, we performed an in vitro kinase assay using recombinant His-HSF1 and GST-DYRK2. We found that DYRK2-WT, but not its kinase-dead version, phosphorylates His-HSF1 at S326 and S320 in vitro (Fig. 1C). Furthermore, incubation of recombinant GST-DYRK2-AS with a PP1 inhibitor inhibited the phosphorylation of His-HSF1 on both S326 and S320, whereas as expected, the phosphorylation mediated by GST-DYRK2-WT was not affected by the PP1 inhibitor (Fig. S1C). On the other hand, harmine inhibited the phosphorylation of His-HSF1 mediated by both GST-DYRK2-WT and GST-DYRK2-AS (Fig. S1D). Mass spectrometry analysis of a sample taken at an early time point (5 min) from the in vitro reaction showed that HSF1 was further phosphorylated at S307, T323 and S363 (Fig. S1E) although these sites could not be validated in cells due to the lack of specific working antibodies. Together, these results show that DYRK2 phosphorylates HSF1 in cells and in vitro at S320 and also at S326, which is critical for HSF1 activation.

Next, to test whether endogenous DYRK2 plays a role in HSF1 activation by proteotoxic stress, we used the prototypical HSF1 inducer heat shock (HS), in the absence or in the presence of harmine, observing that the inhibitor clearly impaired the phosphorylation of HSF1 upon HS (Fig S1F). Although in other cell lines three other kinases (mTOR, MEK1 and p38) can phosphorylate HSF1 at S326 [20, 29, 30], we have previously showed that in TNBC cell lines, MEK1 does not regulate HSF1 [30]. To compare the relevance of mTOR and p38 with that of DYRKs in the activation of HSF1 in response to proteotoxic stress in TNBC, we compared the effect of the DYRK inhibitor harmine, against the p38 inhibitor SB202190 and the mTOR inhibitor rapamycin at impairing HSF1 phosphorylation induced by HS. Our results demonstrate that of the inhibitors and concentrations tested, the DYRK inhibitor harmine is the most effective in impairing HSF1 phosphorylation (Figs. 1D and S1G) in TNBC cells.

### DYRK2 interacts with HSF1 via two domains

**Fig. 2:**
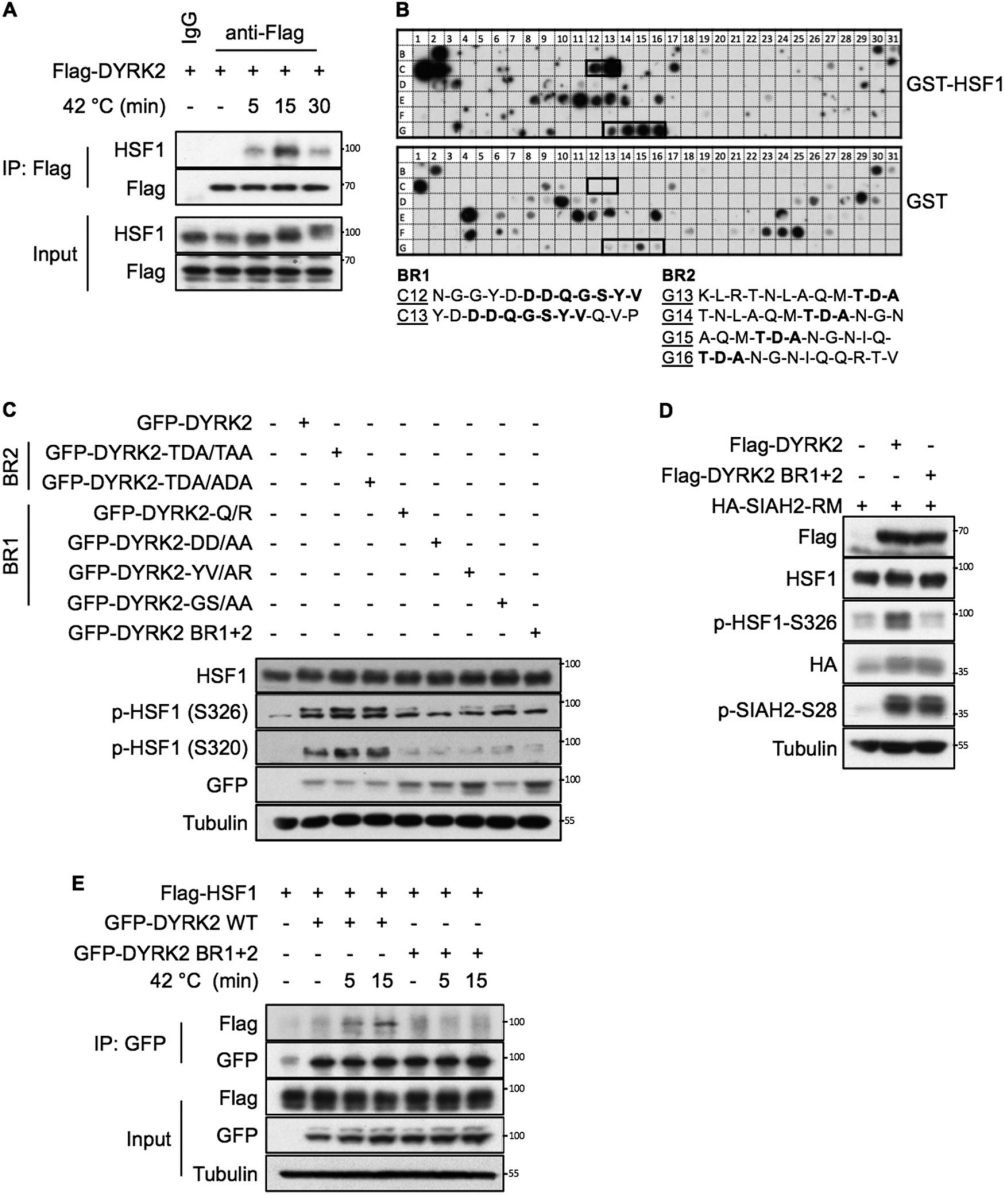
DYRK2 interacts with HSF1 via two domains. **A** 293T cells were transfected with the indicated plasmids. After 48 h, cells were incubated at 42 °C for the indicated periods of time. A fraction of the cell lysates was tested for the correct expression of the transfected DYRK2 (Input), while the remaining extracts were used for immunoprecipitation with either anti-Flag antibodies or with the matched IgG. After elution of bound proteins in 1X SDS sample buffer, coprecipitated HSF1 was visualised by immunoblotting. **B** A peptide array library covering the complete sequence of DYRK2 was incubated with recombinant GST-HSF1 protein (*upper panel*) or a GST control protein (lower panel) and the bound HSF1 protein was revealed by immunoblotting against GST as shown. The specific positive binding regions for HSF1 were indicated with a black box. Sequences of DYRK2 peptides within the binding region 1 (BR1) or binding region 2 (BR2) interacting with HSF1 are shown in bold. **C** 293T cells were transiently transfected with the GFP-tagged versions of either DYRK2-WT or DYRK2 constructs harbouring mutations on the BR1 or BR2 as indicated, or a DYRK2 YV/AR-T/A mutant harbouring mutations in both regions (DYRK2 BR1 + 2). After 48 h, cells were lysed and the levels of endogenous HSF1 and phospho-HSF1 were analysed as indicated. **D** 293T cells were transiently transfected with the Flag-tagged versions of either DYRK2-WT or DYRK2 BR1 + 2, together with an inactive form of SIAH2 (HA-SIAH2-RM). After 48 h, cells were lysed and the levels of the indicated proteins were analysed as indicated. **E** 293T cells were transfected with the indicated plasmids. After 48 h, cells were incubated at 42 °C for the indicated periods of time. A fraction of the cell lysates was tested for the correct expression of the transfected proteins (input), while the remaining extracts were used for immunoprecipitation with anti-GFP antibodies. After elution of bound proteins in 1X SDS sample buffer, coprecipitated HSF1 was visualised by immunoblotting using an anti-Flag antibody as shown. See also Fig. S2.

Our results provide evidence for a functional interaction between DYRK2 and HSF1. In order to test whether DYRK2 and HSF1 physically interact in cells, we performed co-immunoprecipitation assays. The interaction between the two proteins, which was not easily detectable at basal conditions, was increased upon exposure to proteotoxic stress (Figs. 2A and S2A). This weak interaction might reflect a transient binding between DYRK2 and HSF1, which is a common occurrence for kinases and their substrates. In order to map the interaction site(s), we used an in vitro interaction peptide array. By incubating recombinant GST-HSF1 with a membrane containing an array of overlapping peptides covering the entire DYRK2 protein, we identified two potential binding regions (Fig. 2B) within conserved amino acid sequences (Fig. S2B). Binding region 1 (BR1) with sequence DDQGSYV, is located in a surface-exposed loop, and binding region 2 (BR2) with sequence TDA, falls within the unstructured C-terminus of DYRK2 (Fig. S2C). To validate the functional relevance of these two regions, we created DYRK2 constructs harbouring mutations either in BR1 or BR2, or in both simultaneously (BR1 + 2). Based on our results, BR1 appeared to be more important for the ability of DYRK2 to phosphorylate HSF1 (Fig. 2C). Furthermore, when both DYRK2 binding regions were mutated, HSF1 phosphorylation was strongly reduced on both S320 and S326, but notably, the ability of DYRK2 to phosphorylate SIAH2 [31], another known substrate, remained intact (Fig. 2D). This observation suggests that these two regions are not important for general DYRK2 kinase activity, but rather for its specific interaction with HSF1. In agreement, the DYRK2 BR1 + 2 mutant did not interact with HSF1 (Fig. 2E).

### DYRK2 promotes HSF1 nuclear stability

**Fig. 3:**
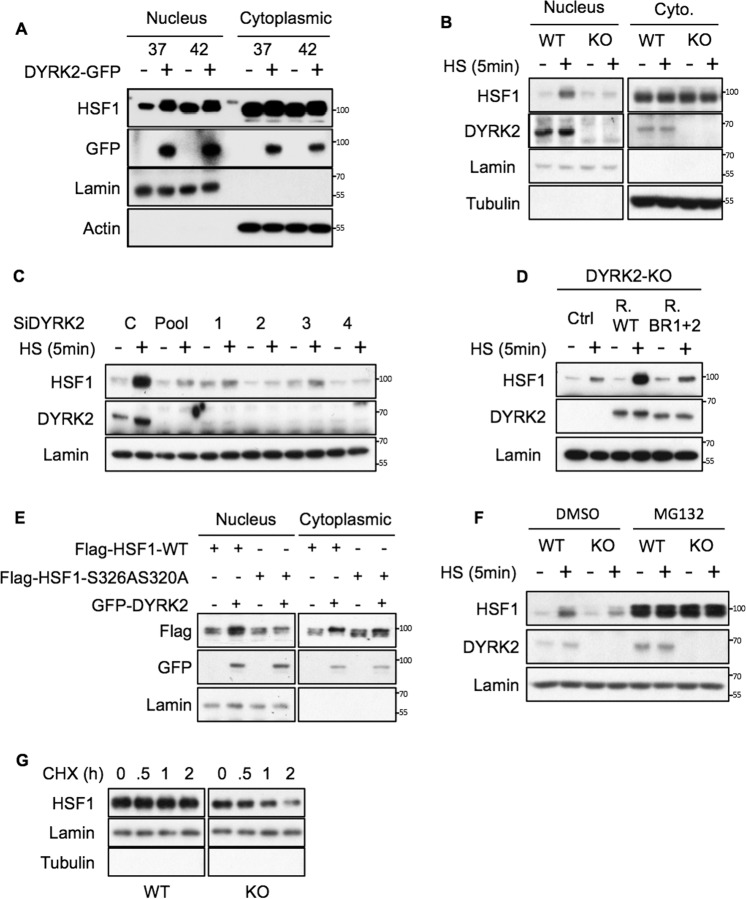
DYRK2 promotes HSF1 nuclear stability. **A** 293T cells were transfected with either empty vector or GFP-DYRK2. After 48 h, cells were incubated at 37 or 42 °C for 5 min. Nuclear and cytoplasmic fractions were analysed by western blot for the levels of the indicated proteins. The corresponding quantifications of HSF1 protein levels are shown in Supplementary Fig. S3A. **B** Control (WT) and CRISPR-mediated DYRK2-KO MDA-MB-468 cells were incubated at 37 or at 42 °C for 5 min. Nuclear and cytoplasmic fractions were analysed by western blot for the levels of the indicated proteins. The corresponding quantifications of HSF1 protein levels are shown in Supplementary Fig. S3B. **C** MDA-MB-468 cells were transfected with either siControl or siDYRK2 (pool) or individual siDYRK2 (1–4). After 48 h, cells were incubated at 37 or at 42 °C for 5 min. Nuclear fractions were analysed by western blot for the levels of the indicated proteins. The corresponding quantifications of HSF1 protein levels are shown in Supplementary Fig. S3E. **D** DYRK2-KO MDA-MB-468 cells transduced with virus encoding for either the empty vector (Ctrl), DYRK2-WT (R. WT) or the DYRK2 HSF1-interaction deficient mutant (R. BR1 + 2) were incubated at 37 or at 42 °C for 5 min. Nuclear fractions were analysed by western blot for the levels of the indicated proteins. The corresponding quantifications of HSF1 protein levels are shown in Supplementary Fig. S3F. **E** 293T cells were transfected with either Flag-tagged HSF1 or with Flag-tagged HSF1 mutant were S320 and S326 were mutated to alanine (S326AS320A) in combination with either empty plasmid or GFP-DYRK2 as indicated. After 48 h, cells were lysed and nuclear and cytosolic fractions were analysed for the levels of the indicated proteins by western blotting. The corresponding quantifications of HSF1 protein levels are shown in Supplementary Fig. S3G. **F** Control (WT) and CRISPR-mediated DYRK2-KO MDA-MB-468 cells were incubated with either DMSO or MG132 (10 µM). After 1 h, cells were incubated at 37 or at 42 °C for 5 min. Nuclear and cytoplasmic fractions were analysed by western blot for the levels of the indicated proteins. The corresponding quantifications of HSF1 protein levels are shown in Supplementary Fig. S3K. **G** Control (WT) and CRISPR-mediated DYRK2-KO MDA-MB-468 cells were incubated with either DMSO (0) or Cycloheximide (10 µM) for the indicated times. Nuclear fractions were analysed by western blot for the levels of HSF1. The corresponding quantifications of HSF1 protein levels are shown in Supplementary Fig. S3L. See also Fig. S3.

Phosphorylation of HSF1 is known to regulate its stability [18, 20, 31]. Phosphorylation at Ser326 and S320 has been involved in promoting HSF1 nuclear accumulation [18, 20], and nuclear HSF1 is the active form. Therefore, we hypothesise that DYRK2-mediated phosphorylation of those two sites might increase HSF1 nuclear levels. To test this, we first overexpressed DYRK2, and looked at the levels of nuclear HSF1 at basal and after HS conditions (Fig. 3A and Fig. S3A). Our results show that DYRK2 overexpression promotes nuclear HSF1 accumulation. To further corroborate our findings, we used the opposite approach, employing stable CRISPR-mediated knockout as well as siRNA-mediated knockdown of DYRK2 to study the effect of DYRK2 depletion on HSF1 nuclear levels. DYRK2 knockout cells showed an impaired HSF1 nuclear accumulation in response to short exposure (5 min) to HS (Figs. 3B and S3B). This impaired accumulation is less obvious at later time points (Fig. S3C, D). In addition, by using a siRNA pool in combination with four individual siRNAs against DYRK2 we showed that transient DYRK2 depletion also impaired the nuclear accumulation of HSF1 in response to short term HS (Figs. 3C and S3E). In our experiments, we used two independent gRNAs to knockout DYRK2 in two different TNBC cell lines (Fig. S3C and S3D), obtaining in both cases, as with the siDYRK2, similar results suggesting a specific effect. Nevertheless, to further corroborate the on-target effect of DYRK2 on HSF1 nuclear levels, we reconstituted TNBC DYRK2-KO cells with either wild-type (WT) DYRK2 or the DYRK2 mutant that cannot interact with HSF1 (BR1 + 2). As shown in figures 3D and S3F, while WT DYRK2 recovers the stabilisation of nuclear HSF1 in response to HS, the BR1 + 2 mutant does not.

To directly test whether the effect of DYRK on HSF1 nuclear accumulation depends on its phosphorylation at S326 and S320, we compared the effect of DYRK2 on the nuclear accumulation of either HSF1 WT or a HSF1 phospho-deficient mutant where both S326 and S320 were mutated to alanine (S326A, S320A) (Figs. 3E and S3G). Our results show that the phosphorylation-deficient mutant shows an impaired accumulation in the nucleus in response to DYRK2 overexpression, suggesting that those two sites are important for the effect of DYRK2 on HSF1 nuclear levels.

Although it has been shown that HSF1 nuclear stability is regulated by the proteasome [32], HSF1 nuclear accumulation in response to HS is still widely considered a consequence of its nuclear translocation. However, in our experiments, we noticed that HSF1 early nuclear accumulation did not have associated any significant cytosolic reduction (Fig. S3H). We thus hypothesised that a reduction of HSF1 nuclear degradation rather than nuclear translocation was responsible for its early accumulation in response to HS. To test whether the proteasome or the autophagosome-lysosomal degradation pathways were involved, we used drugs that inhibit those pathways. While bafilomycin A1 (autophagy/Lysosome inhibitor) did not affect HSF1 nuclear levels (Fig. S3I), low doses of proteasome inhibitor MG132 were able to rapidly stabilise nuclear HSF1 without reducing the cytosolic pool (Fig. S3J). Our results support the hypothesis that the early accumulation of nuclear HSF1 in response to HS depends on the proteasome, suggesting that there is a nuclear pool of HSF1 that is constantly being degraded and that in response to HS gets stabilised (without involving nuclear transport).

Based on this, we tested whether the effect of DYRK2 on nuclear HSF1 was affected by the presence of MG132. As shown in figures 3F and S3K, MG132 recovers the levels of nuclear HSF1 in DYRK2 depleted cells, suggesting that the effect is in fact linked to the proteasome.

To directly assess the effect of DYRK2 on HSF1 nuclear stability, we compared the HSF1 nuclear levels upon cycloheximide treatment in control and DYRK2 knocked-out TNBC cells. In cycloheximide chase experiments, DYRK2 depleted cells had reduced levels of nuclear HSF1 with time, further suggesting that DYRK2 is involved in HSF1 nuclear stability (Figs. 3G and S3L).

### DYRK2 affects the expression levels of the HSF1 target gene *HSP70*

**Fig. 4:**
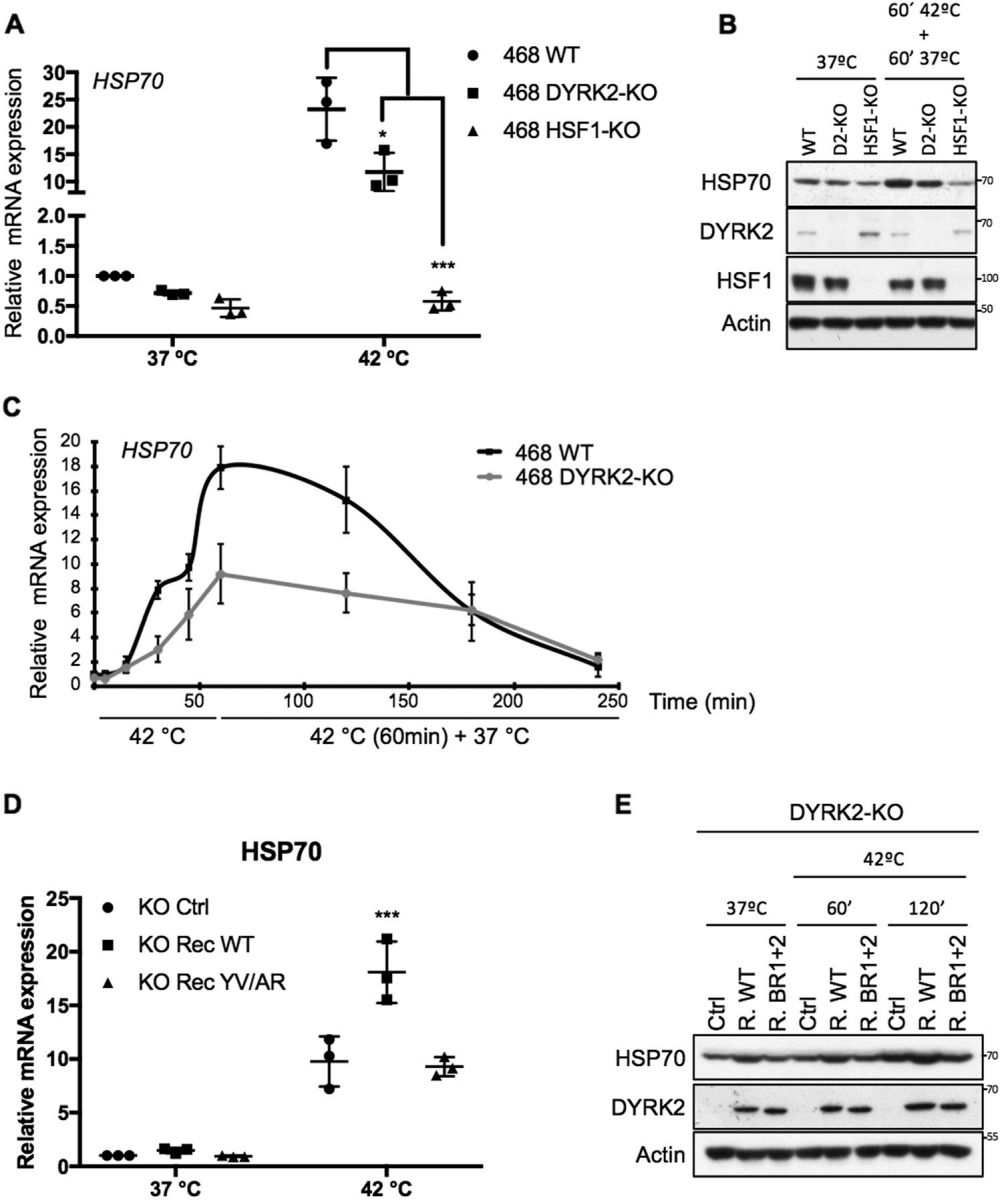
DYRK2 affects the expression levels of the HSF1 target gene *HSP70*. **A** Control (WT), DYRK2-KO or HSF1-KO MDA-MB-468 cells were incubated at 37 or 42 °C for 1 h. The mRNA levels for *HSP70* (*HSPA1A*) were quantified using real-time PCR. The data were normalised using β-actin as an internal control. Data represent means ± SD (*n* = 3) and are expressed relative to the control sample levels at 37 °C. *P ≤ 0.05, **P ≤ 0.01, ***P ≤ 0.001, **B** Control (WT), DYRK2-KO or HSF1-KO MDA-MB-468 cells were incubated at 37 or at 42 °C for 1 h followed by another hour at 37 °C. Cells were lysed and the levels of the indicated proteins were analysed by western blot. The corresponding quantifications of HSP70 protein levels are shown in Supplementary Fig. S4B. **C** Control (WT) and DYRK2-KO MDA-MB-468 cells were incubated at 37 °C, at 42 °C for 5, 15, 30 or 60 min, or at 42 °C for 60 min plus recovery time at 37 °C (up to 180 min). After that, mRNA levels for *HSP70* (*HSPA1A*) were quantified using real-time PCR. The data were normalised using β-actin as an internal control. Data represent means ± SD (*n* = 3) and are expressed relative to the control samples at 37 °C. **D** DYRK2-KO MDA-MB-468 infected with virus encoding for either empty vector (Ctrl), DYRK2-WT (R. WT) or the DYRK2 HSF1-interaction deficient mutant (R. BR1 + 2) were incubated at 37 °C or at 42 °C for 60 min. After that, mRNA levels for HSP70 (HSPA1A) were quantified using real-time PCR. The data were normalised using β-actin as an internal control. Data represent means ± SD (*n* = 3) and are expressed relative to the control samples at 37 °C. *P ≤ 0.05, **P ≤ 0.01, ***P ≤ 0.001. **E** DYRK2-KO MDA-MB-468 infected with virus encoding for either empty vector (Ctrl), DYRK2-WT (R. WT) or the DYRK2 HSF1-interaction deficient mutant (R. BR1 + 2) were incubated at 37 °C or at 42 °C for either 60 or 120 min. Cells were lysed and the levels of the indicated proteins were analysed by western blot. The corresponding quantifications of HSP70 protein levels are shown in Supplementary Fig. S4D. See also Fig. S4.

Based on its positive effect on the levels of nuclear (active) HSF1, we hypothesised that DYRK2 could be promoting HSF1 transcriptional activity. To test this hypothesis, we compared the levels of the prototypic HSF1 target gene, HS protein 70 (*HSP70*), in TNBC cells with and without DYRK2. DYRK2 knockout reduced the inducible *HSP70* mRNA and protein levels in response to HS (Figs. 4A–C and S4A, B) and also to other proteotoxic stress inducers, such as bortezomib (Fig. S4C); quantitative real-time PCR revealed that in all cases, this reduction was by ~50%. Similarly, and as expected, HSF1 knockout strongly reduced the expression of HSP70, as well as its protein levels (Figs. 4A, B and S4A, B). Importantly, the reduction on both HSP70 expression and protein levels observed in TNBC DYRK2-KO cells was recovered by reconstituting them with the WT form of DYRK2, but not with the DYRK2-HSF1 interaction deficient mutant (BR1 + 2) (Figs. 4D, E and S4D).

### DYRK2 reduces sensitivity to proteotoxic stress via HSF1

**Fig. 5:**
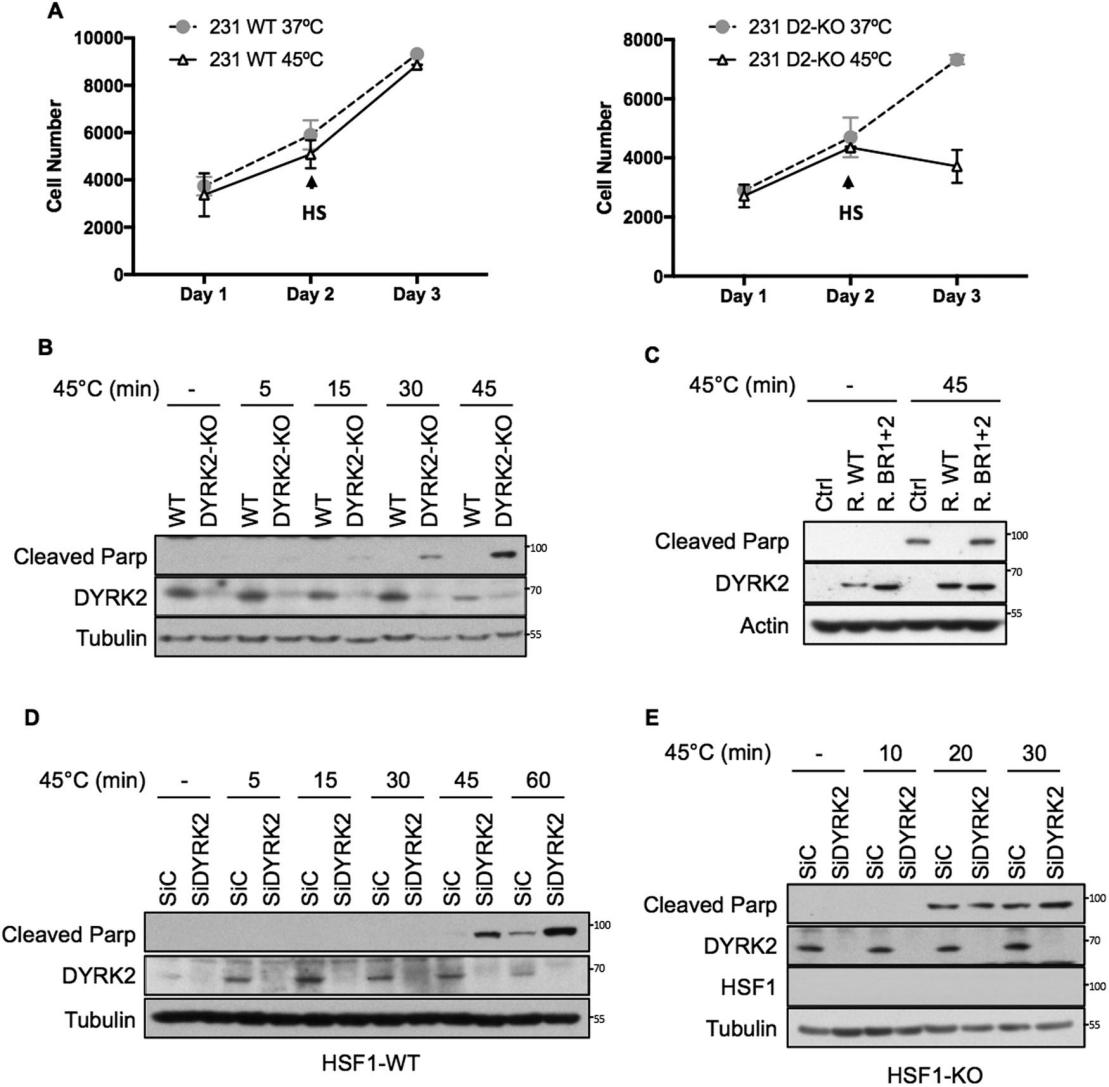
DYRK2 reduces sensitivity to proteotoxic stress via HSF1. **A** Equal number of MDA-MB-468 WT (Left panel) or DYRK2-KO (Right panel) were seeded. After 24 h, cells were exposed to 45 °C (HS) for 45 min followed by recovery at 37 °C (cells labelled as 45 °C) or they were left at 37 °C (cells labelled as 37 °C). Number of cells at each point were analysed using the Alamar Blue assay. Data represent means ± SD (*n* = 3). **B** Control (WT) and DYRK2-KO MDA-MB-468 cells were incubated at 37 °C (−) or at 45 °C for the indicated times, followed by recovery at 37 °C. On the next day, cells were lysed and the levels of apoptosis were analysed by western blotting using an antibody that recognises cleaved PARP. The corresponding quantifications of PARP protein levels are shown in Supplementary Fig. S5A. **C** DYRK2-KO MDA-MB-468 infected with virus encoding for either empty vector (Ctrl), DYRK2-WT (R. WT) or the DYRK2 HSF1-interaction deficient mutant (R. BR1 + 2) were incubated at 37 °C or at 45 °C for 45 min followed by recovery at 37 °C. On the next day, cells were lysed and the levels of apoptosis were analysed by western blotting using an antibody that recognises cleaved PARP. The corresponding quantifications of PARP protein levels are shown in Supplementary Fig. S5B. MDA-MB-468 (**D**) or HSF1-KO MDA-MB-468 (**E**) cells transfected with either siControl or siDYRK2 were incubated at 37 °C or at 45 °C for the indicated times, followed by recovery at 37 °C. On the next day, cells were lysed and the levels of apoptosis were analysed by western blotting using an antibody that recognises cleaved parp. The corresponding quantifications of PARP protein levels are shown in Supplementary Fig. S5C, D. See also Fig. S5.

Based on our data highlighting the importance of DYRK2 for HSF1 function in TNBC cells and on the well-characterised protective role of HSF1 against proteotoxicity, we hypothesised that DYRK2 might protect TNBC cells against proteotoxic stress. To assess the functional significance of the reduced HSF1 activity in DYRK2-impaired TNBC cells, we exposed cells to 45 °C (mild/severe HS) which is a widely used stress to measure thermo-tolerance, and as a proxy for HSF1 activity. DYRK2 depletion sensitised TNBC cells to HS, as measured by cell viability (Fig. 5A) and by the levels of PARP cleavage, a marker of apoptosis (Figs. 5B and S5A). Importantly, the levels of apoptosis were reduced by reconstituting the DYRK2 knockout cells with the WT form of DYRK2, but not by reconstituting them with the DYRK2-HSF1 interaction deficient mutant (BR1 + 2) (Figs. 5C and S5B). In addition, the effect of DYRK2 knockdown on the enhanced sensitivity to proteotoxic stress in WT cells (Figs. 5D and S5C) was largely abolished in TNBC HSF1-KO cells (Figs. 5E and S5D), confirming the importance of HSF1 for the cytoprotective role of DYRK2 in response to HS.

As DYRK2 has also been involved in the response to DNA damage [33], we also tested the sensitivity of WT and DYRK2-KO TNBC cells to doxorubicin and paclitaxel (Taxol), two chemotherapeutic drugs used in the clinic. As shown in figure S5E, F, DYRK2-KO cells were not more sensitive than the WT cells to these drugs, suggesting that the previously observed increased sensitivity to HS is not a general response to stress, but rather specific to proteotoxic stress. In agreement, TNBC DYRK2-KO cells are also more sensitive to the proteotoxic stress inducer Bortezomib [34].

### DYRK2 levels correlate with HSF1 nuclear levels, and associate with prognosis and tumour recurrence in tissue from TNBC patients

**Fig. 6:**
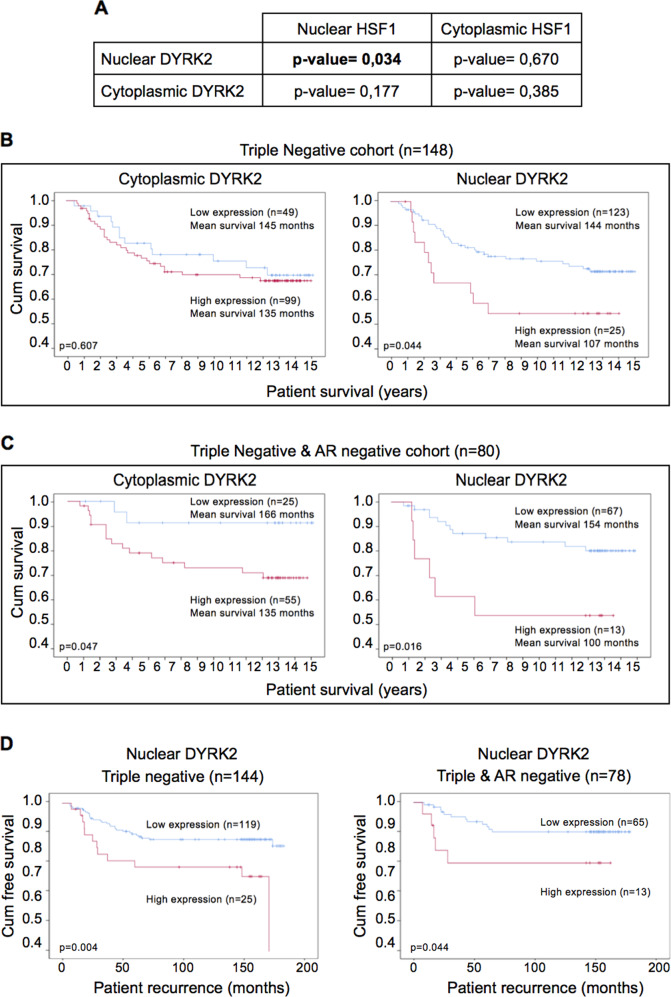
DYRK2 levels correlate with HSF1 nuclear levels and associates with poor prognosis and tumour recurrence in tissue from TNBC patients. **A** Chi-Square test correlations between nuclear/cytoplasmic HSF1, and nuclear/cytoplasmic DYRK2 in triple-negative breast cancer. Relationship between either cytoplasmic DYRK2 levels (left panel) or nuclear DYRK2 levels (right panel) and cancer-specific survival in (**B**) patients with triple-negative invasive ductal breast cancer, and (**C**) patients with triple-negative and AR-negative invasive ductal breast cancer. **D** Relationship between nuclear DYRK2 levels in tumour cells and time to recurrence in patients with triple negative (left panel) or triple negative and AR-negative (right panel) invasive ductal breast cancer. See also Fig. S6.

The data so far show that DYRK2 regulates HSF1 nuclear levels in TNBC cell lines. Next, as a first approximation to address if DYRK2 regulates HSF1 in vivo, we asked whether the levels of DYRK2 correlate with HSF1 levels in tumour tissue from TNBC patients. To answer this question, immunohistochemistry (IHC) was employed and the protein levels of DYRK2 and HSF1 in both the cytoplasm and nucleus were assessed separately (antibodies validation and representative figures in figure S6A, B, C). In a cohort of tissue samples from 102 TNBC patients, a weak correlation was observed between cytoplasmic DYRK2 and cytoplasmic HSF1, and between nuclear DYRK2 and nuclear HSF1 (Fig. S6D). The samples were then dichotomised into high and low levels groups for DYRK2 and HSF1 in relation to a cut-off, determined using a receiver operating characteristic (ROC) curve, with cancer death as an endpoint. Using this method, the cut-off was defined as 145 for cytoplasmic DYRK2; 145 for nuclear DYRK2; 100 for cytoplasmic HSF1; and 160 for nuclear HSF1. When analysed, no association by chi-square test was observed between cytoplasmic DYRK2 and HSF1 (*p* = 0.385). By contrast, a significant correlation was observed between nuclear DYRK2 and nuclear HSF1 (*p* = 0.034) (Fig. 6A), suggesting that high nuclear levels of DYRK2 correlate with high nuclear HSF1 levels in triple-negative disease.

By interrogating publicly available datasets, we show that, as recently reported [35], DYRK2 mRNA levels are often higher in tumour than in normal tissue, correlating in some cases with poor patient survival (Fig. S6E). We have recently showed that DYRK2 protein levels are higher in TNBC tumours than in adjacent normal tissue [36], however, the relationship between DYRK2 protein levels and patient survival in different tumour types has not been properly studied. Based on the observed novel correlation between nuclear DYRK2 and nuclear HSF1 in TNBC human samples and on the known association of nuclear HSF1 with poor prognosis [10, 13], we hypothesised that high levels of nuclear DYRK2 would also associate with poor prognosis in TNBC. To test this hypothesis, we utilised a cohort of 750 breast cancer samples that had available a previously constructed tissue microarray, including 148 TNBC (tissue microarrays (TMA) information in Fig. S6F). Protein expression in both the tumour cell cytoplasm and tumour cell nucleus was categorised as either “low” or “high”, in relation to a cut-off as previously indicated. DYRK2 protein levels were assessed in three individuate TMA cores and mean weighted histoscore units (WHU) employed for analysis (representative figures with high and low DYRK2 levels in Fig. S6C). In the full cohort, DYRK2 levels did not associate with survival (Fig. S6G). However, in TNBC samples, high nuclear DYRK2 levels significantly associated with reduced cancer-specific survival (Fig. 6B) but not with overall survival (Fig. S6H). Notably, the strength of this association was highest in the TNBC subgroup of patients with ER, PR, HER2 and AR-negative disease (Fig. 6C). In TN-AR-negative samples, nuclear DYRK2 was observed as an independent factor in Cox Regression multivariate analysis when combined with other clinical parameters (Fig. S6I). Moreover, high nuclear DYRK2 levels were associated with shorter time to recurrence in both TNBC and TN-AR-negative breast cancer (Fig. 6D).

These results established DYRK2 as a potential prognostic factor and promising novel therapeutic target in TNBC, especially in the TN/AR-negative subgroup of patients, for whom there is no targeted therapy available. Interestingly, several of the commonly used TNBC cell models (including the two cell lines used in this study, MDA-MB-231 and MDA-MB-468) are also AR-negative [37].

## Discussion

Cancer cells often rely on protein degradation and folding pathways (i.e. proteasome, autophagy and HSF1 pathways) to survive the high proteotoxic stress levels associated with malignancy. The identification of new ways to target these pathways, ideally simultaneously, might therefore provide new and generalised therapeutic opportunities to target cancer cells independently of their individual genetic lesions responsible for cancer initiation. Indeed, the HSF1 pathway represents an attractive therapeutic target as it not only allows cancer cells to survive aneuploidy-induced proteotoxic stress, but it also plays an important role in cancer progression and chemoresistance [8, 10, 13, 38, 39]. However, developing specific HSF1 inhibitors has proven to be challenging, and thus, an alternative approach is to understand and to target HSF1 upstream regulatory pathways. In this study, we shed light on the regulation of the HSF1 pathway by the kinase DYRK2. We propose that DYRK2 regulates HSF1 nuclear levels and it is the main kinase regulating HSF1 activation in TNBC, and the first kinase described to phosphorylate HSF1 at both S326 and S320 sites. Remarkably, DYRK2 depletion is sufficient to impair HSF1 nuclear levels and HSP70 expression, increasing TNBC cell sensitivity towards proteotoxic stress. This suggests that although other kinases might also regulate HSF1 activity, they cannot completely compensate for the absence of DYRK2.

Our data show that DYRK2 activates the pro-survival HSF1 pathway providing a support mechanism and a survival advantage to cancer cells. Importantly, this new link between DYRK2 and HSF1 appears to be relevant in TNBC patients as DYRK2 levels correlate positively with HSF1 nuclear levels, and negatively associate with cancer-specific survival and time to recurrence, supporting a pro-tumoural role for DYRK2 in TNBC. Furthermore, our data suggest that nuclear DYRK2 might be the pool responsible for its HSF1-dependent tumour promoter role in TNBC.

Although it has been suggested that DYRK2 might have both a “tumour suppressor” and a “tumour promoter” role [34, 40–44], our data support the hypothesis that in TNBC DYRK2 acts as a “tumour promoter”. Furthermore, our results suggest that DYRK2 may be both a prognostic biomarker and a potential therapeutic target, which will be especially relevant in TN-AR-negative breast cancer patients, for whom there is no targeted therapy available. The development of new specific DYRK2 inhibitors and their validation in vivo (i.e. using xenograft and genetic models) is needed to further substantiate this hypothesis. In this context, it is encouraging to see the results from two recent studies showing that treatment with the DYRK2 inhibitors curcumin and LDN192960 impaired growth of established TNBC tumours [36, 40]

Our data, together with previous reports showing that DYRK2 controls proteasome activity [34], presents DYRK2 as a major apical positive regulator of two main pathways involved in alleviation of proteotoxic stress in cancer cells, and thus two main mechanisms by which aneuploid cells adapt, survive and become malignant. Thus, DYRK2 inhibition, by impairing both supporting mechanisms, might be an excellent way to tackle not only TNBC cells, but also other aneuploid cancer cells that depend on such support mechanisms, independently on their genetic background.

## Material and methods

### Cell culture

MDA-MB-231 were obtained from the culture collection at Public Health England. 293T and MDA-MB-468 cell lines were obtained from ATCC. All cell lines were grown in DMEM containing 10% FBS at 37 °C and 5% CO_2_. Cells were routinely tested for mycoplasma and those that were not recently bought, were authenticated by SRT profile before submission. Cells are passaged once 70–90% confluency is reached, and are maintained in culture for no more than 20 passages. Freshly thawed cells are passaged 2–3 times before used.

### Antibodies, plasmids and reagents

Antibodies recognising Flag (F1804) were obtained from Sigma-Aldrich (Dorset, UK); anti-HA (sc-805), anti-Lamin B2 (sc-56147) and anti-Tubulin (sc-8035) were obtained from Santa Cruz Biotechnology (Dallas, Texas, USA). Anti-GFP anti-DYRK2 were obtained from the MRC Protein Phosphorylation and Ubiquitination Unit, (School of Life Science, University of Dundee, UK); anti-cleaved PARP (9546S) and anti-DYRK2 (8143) were obtained from Cell Signalling (MA, USA); anti-HSF1 (ADI-SPA-901-D) was obtained from Enzo Life Science (NY, USA); anti-phospho-HSF1-Ser 326 (ab115702), anti-phospo-HSF1-Ser 320 (ab76183) and anti-GST-HRP (ab3416) were obtained from Abcam (Cambridge, UK). HRP-conjugated secondary antibodies (31430 and 31460) and Dynabeads™ Protein G (10004D) were obtained from Life Technologies (Carlsbad, California, USA).

Expression vectors for DYRK2-GFP, DYRK2-KD-GFP, DYRK2-Flag and SIAH2-RM-HA were a gift from Marco A. Calzado (University of Cordoba, Spain); HSF1-GFP have been already described [30]. All gRNAS were cloned into pLentiCRISPr-V2, which was a gift from Feng Zhang (Addgene plasmid #52961). Point mutants were produced by conventional point mutagenesis. The DYRK2-analogue sensitive was obtained by mutating the gatekeeper residue F228 to Alanine. His-HSF1 has already been described [30] and GST-DYRK2 was obtained from the MRC Protein Phosphorylation and Ubiquitination Unit (School of Life Science, University of Dundee, UK). DYRK2-WT and DYRK2-BR1 + 2 were cloned into the lentiviral 290-puro plasmid [45] obtaining a puro resistant construct that was used for reconstitution experiments.

The siRNAs used against DYRK2 were the SMART pool: ON-Target Plus, and its four individual siRNAs all from Dharmacon (CO, USA).

Bortezomib (sc-217785) was obtained from Santa Cruz Biotechnology. Harmine (5075) was obtained from Tocris Bioscience (Bristol, UK), PP1 inhibitors (17860, 10954 and 13330) were obtained from Cayman Chemicals (MI, USA). The p38 inhibitor SB202190 (1073) was obtained from SYNkinase (3052, Australia) and the mTOR inhibitor Rapamycin (553210) was obtained from Sigma-Aldrich.

### Quantitative real-time PCR (rt-qPCR)

RNA was extracted using RNeasy kit (QIAGEN). Overall, 500 ng of RNA per sample was reverse-transcribed to cDNA using Omniscript RT kit (QIAGEN) supplemented with RNase inhibitor (QIAGEN) according to the manufacturer’s instructions. Resulting cDNA was analysed using TaqMan Universal Master Mix II (Life technologies). Gene expression was determined using an Applied Biosystems 7300 Real-Time PCR system by the comparative ΔΔCT method. All experiments were performed at least in triplicates and data were normalised to the housekeeping gene β-actin. Probes were obtained from Applied Biosystems. When applicable, the differences between groups were determined by two-way ANOVA. Analyses were performed using GraphPad Prism (GraphPad Software); a *P* value of <0.05 was considered significant. *P ≤ 0.05, **P ≤ 0.01, ***P ≤ 0.001.

### Cell transfections

On the day prior to transfection, cells were plated to the required cell density (70–90% confluency). Lipofectamine 2000 and Lipofectamine RNAiMAX (Invitrogen) were used for plasmid DNA and siRNA, respectively. The plasmid DNA/siRNA and lipofectamine were individually diluted in Optimem (Gibco) and incubated for 10 min at room temperature. Diluted DNA/siRNA was added to the diluted Lipofectamine solution (1:1 ratio) and further incubated for 15 min. DNA-lipid complex was added to the cells and incubated overnight in a humidified incubator at 37 °C and 5% CO_2_. The next morning, the medium was replaced with fresh medium and cells were incubated 36 h more prior lysis.

### Cell viability assays

Equal number of the different cell lines were seeded. After treatment (either HS or chemotherapeutic drugs) the number of metabolically active cells were measured using the Alamar Blue assay (Thermo Fisher Scientific) following the manufacturer’s instructions.

### Lentivirus production and cell transduction

293T cells were transfected using Lipofectamine 2000 (Invitrogen) with the empty vector (290-pHAGE-hEF1a CAR-PGK Puro) or the lentiviral DYRK2-WT, or DYRK2 mutant (BR1 + 2) together with the packaging vectors (pMDLg/pRRE, pRSV-Rev and pHCMVG) and cultivated in OptiMEM medium (Invitrogen). The next day the cells were further grown in DMEM complete medium and 1 day later the lentivirus-containing supernatant was collected, filtered and used to transduce cells.

Cells were incubated with the media-containing virus complemented with Polybrene (8 μg/ml) for 16 h, followed by a medium exchange. Transduced cells were selected by further growth in the presence of 2 μg/ml puromycin; the surviving cells were tested by immunoblotting for adequate DYRK2 expression.

### Cell lysis protocol and western blotting

Cells were washed and harvested in ice-cold phosphate-buffered saline (PBS) and lysed in either SDS buffer or RIPA buffer (50 mM Tris-HCl pH 7.5, 150 mM NaCl, 2 mM EDTA, 1% NP40, 0.5% sodium deoxycholate, 0.5 mM Na3VO4, 50 mM NaF, 2 μg/ml leupeptin, 2 μg/ml aprotinin, 0.05 mM pefabloc). Cells directly lysed in SDS were boiled for 2 min, sonicated and boiled again for another 5 min. Cells lysed in RIPA buffer were sonicated and lysates were cleared by centrifugation for 15 min at 4 °C. Protein concentration was established using the BCA assay (Pierce). Supernatant was mixed with SDS sample buffer and boiled for 5 min. Equal amounts of protein were separated by SDS-PAGE, followed by semidry blotting to a polyvinylidene difluoride membrane (Thermo Scientific). After blocking of the membrane with 5% (w/v) TBST non-fat dry milk, primary antibodies were added. Appropriate secondary antibodies coupled to horseradish peroxidase were detected by enhanced chemiluminescence using Clarity™ Western ECL Blotting Substrate (BIO-Rad).

### Subcellular fractionation (nuclear/cytoplasmic separation)

Cells were washed and harvested with ice-cold PBS. Pelleted cells were resuspended in 400 μl of low-salt buffer A (10 mM Hepes/KOH pH 7.9, 10 mM KCL, 0.1 mM EDTA, 0.1 mM EGTA, 1 mM β-Mercaptoethanol). After incubation for 10 min on ice, 10 μl of 10% NP-40 was added and cells were lysed by gently vortexing. The homogenate was centrifuged for 10 s at 13,200 rpm in a microfuge. The supernatant representing the cytoplasmic fraction was collected and the pellet containing the cell nuclei was washed 4 additional times in buffer A, then resuspended in 100 μl high-salt buffer B (20 mM Hepes/KOH pH 7.9, 400 mM NaCL, 1 mM EDTA, 1 mM EGTA, 1 mM β-Mercaptoethanol). The lysates were sonicated and centrifuged at 4 °C for 15 min at 13,200 rpm. The supernatant representing the nuclear fraction was collected. Protease and phosphatase inhibitors were freshly added to both buffers.

### Immunoprecipitation

Cells were washed with PBS and lysed in IP buffer (50 mM Hepes pH 7.5, 50 mM NaCl, 1% (v/v) Triton X-100, 2 mM EDTA, 10 mM sodium fluoride, 0.5 mM sodium orthovanadate, leupeptin (10 µg/ml), aprotinin (10 µg/ml), and 1 mM PMSF), followed by a sonication step. The extract was centrifuged and the supernatant was transferred to a new tube. The immunoprecipitation was performed with 2 μg of precipitating antibodies together with 50 µl of Dynabeads™ Protein G. Tubes were rotated for 30 min on a spinning wheel at 4 °C. The immunoprecipitates were washed 3x with PBS/0,01% Tween-20 and eluted by boiling in 1X SDS sample buffer. Equal amounts of protein were separated by SDS-PAGE.

### CRISPR-edited cell lines

The endogenous DYRK2 or HSF1 genes were knocked out by transfecting cells with pLentiCRISPR-v2 (which codes for Cas9, and a puromycin cassette) containing gRNAs against the first exon of the short DYRK2 isoform or against the fourth exon of HSF1. For MDA-MB-231 cells the gRNA sequence used was GCTTGCCAGTGGTGCCAGAG and for MDA-MB-468 DYRK2-KO cells the target sequence was CGCTCACGGACAGATCCAGG. In addition, we also tested some of our results in MDA-MB-231 cells in which we almost completely remove the DYRK2 ORF by using two gRNAs (N-term sequence GCTTGCCAGTGGTGCCAGAG and C-term sequence GAAGCTGAGCTAGAAGGTGG). For HSF1-KO cells the gRNA sequence used was AAGTACTTCAAGCACAACAA. Control cells were transfected with the empty pLentiCRISPRV2 vector. After transfection, cells were exposed to 2 μg/ml of puromycin for 2 days followed by a medium exchange. Surviving cells were clonally selected (in the case of control cells were used as pool population) by serial dilution, and positive clones were identified by genomic analysis and western blot. At least two clones for each cell line were used for the experiments.

### In vitro kinase assay

Bacterially expressed full length HSF1 His tagged was incubated with GST-DYRK2 in Kinase Buffer (25 mM TRIS pH 7.5, 5 mM β-glycerophosphate, 10 mM MgCl2 and freshly added 2 mM DTT, 0.1 mM Na3VO4, 1 mM ATP) for different periods of time (5–60 min) at 30 °C. The reaction was inactivated by adding SDS loading buffer (50 mM Tris-HCl pH 6.8, 10%, SDS, 40% glycerin, 15% β-mercaptoethanol, 0.1% bromophenol blue) and samples were boiled at 95 °C for 5 min and loaded in SDS-PAGE gels.

### In vitro peptide binding

Overlapping dodecapeptides covering the entire DYRK2 protein were spotted in an automated process on cellulose membranes by using Fmoc-protection chemistry (Proteomics facility, CNB-CSIC, Spain). The membrane was blocked overnight in non-fat milk in TBS buffer and incubated for 6 h with 60 nmol of recombinant GST-HSF1 or GST proteins as a control. After extensive washing, anti-GST antibody coupled to horseradish peroxidase was added for 1 h. After washing, blots were revealed by enhanced chemiluminescence using Clarity™ Western ECL Blotting Substrate (BIO-Rad).

### Mass spectrometry

Purified His-HSF1 (1 ug) was incubated with GST-DYRK2 (20 ng) in 20 uL buffer containing 25 mM Tris-HCl (pH 7.5), 5 mM MgCl2, and 1 mM ATP for 5 min. The mixtures were reduced, alkylated and digested with trypsin (1:100) prepared in 100 mM TEAB buffer (pH 8.5). The mixture was incubated at 37 °C overnight before 8 uL of 10 mM EDTA (in 0.1% TFA) was added. The peptide mixture was then subjected to nano-LC-MS/MS analysis according to a previous report [46]. Database search was carried out using Peaks 7.0 against UniProt-human (version 2018-05-28). False discovery rate was set at 1% (for peptide spectrum matches). The non-modified and modified (methionine oxidation and phosphorylation) peptides from HSF1 detected are shown in blue lines. The putative oxidation sites are labelled in yellow and phosphorylation sites in red.

### Patient cohort and tissue microarrays

Patients presenting with invasive ductal breast cancer at Glasgow Royal Infirmary, Western Infirmary and Stobhill Hospital between 1995 and 1998, with formalin-fixed paraffin-embedded blocks of the primary tumour available for evaluation were studied (*n* = 850). The study was approved by the Research Ethics Committee of the West Glasgow University Hospitals NHS Trust with consent obtained from all subjects. Clinicopathological data including age, tumour size, tumour grade, lymph node status, type of surgery and use of adjuvant treatment (chemotherapy, hormonal based therapy and/ or radiotherapy) were retrieved from the routine reports. Tumour grade was assigned according to the Nottingham Grading System. ER and PR status were assessed on TMAs using IHC with Dako (Glostrup, Denmark) ER antibody and Leica (Wetzlar, Germany) PR antibody. Her2 status was assessed as described [47].

### Tissue analysis

IHC was conducted in triplicate on TMAs. Prior to this, antibody specificity was confirmed using proficient and deficient cell lines (either knockdown or knockout cell lines) (Fig. S6A). TMA sections (2.5 µm) were dewaxed by immersion in histoclear then rehydrated using a series of alcohols. Heat-induced antigen retrieval was performed in a solution of either Tris-EDTA pH9 (DYRK2) or citrate buffer pH 6 (HSF1) after which the sections were incubated in 3% hydrogen peroxide. Non-specific binding was blocked by incubation in 1.5% normal horse serum before being incubated with the optimum concentration of primary antibody overnight at 4 °C. Antibody dilutions were prepared in antibody diluent (Agilent, London, UK). The primary antibodies and concentrations used are as follows; DYRK2 (Abgent AP7534a) at 1:200 and HSF1 (Cell Signalling 4256) at 1:500. Staining was visualised using ImmPRESS^TM^ and ImmPACT^TM^ DAB, (both Vector Labs). Tissue was counterstained using Harris Haematoxylin before being dehydrated and mounted using DPX.

The stained TMA sections were scanned using a Hamamatsu NanoZoomer (Welwyn Garden City, Hertfordshire, UK) at ×20 magnification. Visualisation was carried out using Slidepath Digital Image Hub, version 4.0.1 (Slidepath, Leica Biosystems, Milton Keynes, UK). Protein expression was performed at a magnification of ×400, expression in cytoplasmic and nuclear compartments was assessed separately using the Weighted Histoscore Technique as previously described [48]. The weighted histoscore is performed by assessing the percentage cells with no staining (0), percentage of cells with weak staining (1), percentage of cells with moderate staining (2), and percentage of cells with strong staining (3) for each of the cellular locations. This was then entered into the below formula to provide a score from 0 to 300.

**Total score = (% of unstained tumor cells × 0) + (% of weakly stained tumor cells × 1) + (% of moderately stained tumor cells × 2) + (% of strongly stained tumor cells × 3)**

Where a total score of 0 means tumour tissue is negative for protein expression at that cellular location, and a total score of 300 means tumour tissue strongly expressed protein at that cellular location.

Since each patient was represented by three different cores on the TMA, this was repeated for each core, and mean of these was calculated to provide the score used in the analysis. The weighted histoscore method was employed to assess protein expression (GB), with a second independent observer (JE) scoring 10% of the scores. The results were considered discordant if the histoscores differed by more than 50 WHU.

Protein expression in both the tumour cell cytoplasm and tumour cell nucleus was categorised as either “low” or “high”, in relation to a cut-off. This cut-off was determined using a ROC curve based on survival, with cancer death as an endpoint.

## Supplementary information

Supplementary Figure Legends

Supplementary Figure 1

Supplementary Figure 2

Supplementary Figure 3

Supplementary Figure 4

Supplementary Figure 5

Supplementary Figure 6
