# Supplementary material for: The stress-responsive kinase DYRK2 activates heat shock factor 1 promoting resistance to proteotoxic stress
Source: Cell Death Differ. 2020 Dec 2;28(5):1563–78. doi: 10.1038/s41418-020-00686-8 (PMC8166837; doi:10.1038/s41418-020-00686-8)
Supplement: Supplementary file 1 — Supplementary Figure Legends [file 41418_2020_686_MOESM1_ESM.docx]

**Figure S1. Related to Figure 1. DYRK2 phosphorylates HSF1. A)** Kinase profiling of Harmine at 10 μM was carried out against the panel of 140 kinases at the The International Centre for Protein Kinase Profiling (www.kinase-screen.mrc.ac.uk/). **B)** 293T cells were transiently transfected with empty vector or Flag-tagged DYRK2 as indicated. After 48 hours, cells were treated for a further 3 hours with vehicle or harmine (8 µM). Cells were lysed and the levels of endogenous HSF1 and phospho-HSF1 were analysed as indicated. **C and D)** 20 ng of recombinant GST-DYRK2-WT (*left panel*) or GST-DYRK2-AS (*right panel*) were incubated with His-HSF1 (1 µg) and increasing concentrations of either 1NM-PP1 (C) or hamine (D) in kinase buffer without ATP. After 5 minutes, ATP was added and the reaction incubated at 30°C for 30 min. The reactions were terminated by the addition of SDS gel loading buffer, the proteins were resolved by SDS-PAGE, and the levels of phosphorylated HSF1 were analysed. **E)** Nano-LC-MS/MS analysis of His-HSF1 incubated with GST-DYRK2 for 5 minutes. Database search was carried out using Peaks 7.0 against UniProt-human(version 2018-05-28). False discovery rate was set at 1% (for peptide spectrum matches). The non-modified and modified (methionine oxidation and phosphorylation) peptides from HSF1 detected are shown in blue lines. The putative oxidation sites are labled in yellow and phosphorylation sites in red. **F)** MDA-MB-468 cells were treated with increasing concentrations of harmine. One hour later, cells were incubated at 42 °C. After one hour, cells were lysed in SDS buffer and the levels of the indicated proteins were analysed by western blotting. **G)** MDA-MB-231 cells were treated with either DMSO, the p38 inhibitor SB202190 (10 µM), the mTOR inhibitor rapamycin (30 nM) or harmine (10 µM). One hour later, cells were incubated at 42 °C. After one hour, cells were lysed in SDS buffer and the levels of the indicated proteins were analysed. Upper panel is a representative western blot and the bottom panel shows the quantification of the ratio between the phospho-HSF1 and total HSF1 levels. Data represent means ± SD (n = 3) and are expressed relative to the DMSO treated samples

**Figure S2. Related to Figure 2. DYRK2 interacts with HSF1 via two domains. A)** 293T cells were transfected with the indicated plasmids. After 48 hours, cells were incubated at 42 °C for the indicated periods of time. A fraction of the cell lysates was tested for the correct expression of the transfected proteins (Input), while the remaining extracts were used for immunoprecipitation with anti-HA antibodies. After elution of bound proteins in 1 x SDS sample buffer, coprecipitated HSF1 was visualized by immunoblotting. **B)** Species alignment for the DYRK2 sequence containing the two identified binding regions, BR1 and BR2 (underlined). Asterisks mark conserved residues. **C)** Representation of the 3D structure of DYRK2 obtained for UniProt. The square focused on the DDQGSYV region and shows in blue the position of the different residues.

**Figure S3. Related to Figure 3. DYRK2 promotes HSF1 nuclear stability. A)** Quantification for Fig. 3A of the protein levels of HSF1 against its loading control. Data represent means ± SD (n = 3) and are expressed relative to the 37°C empty control sample. **B)** Quantification for Fig. 3B of the protein levels of HSF1 against its loading control. Data represent means ± SD (n = 4) and are expressed relative to the 37°C WT control sample. **C and D)** Control (WT) and CRISPR-mediated DYRK2-KO MDA-MB-468 (C) and MDA-MB-231 (D) cells were incubated at 37 °C or at 42 °C for the indicated times. Nuclear and cytosolic fractions were analysed by western blot for the levels of the indicated proteins. **E)** Quantification for Fig. 3C of the protein levels of HSF1 against its loading control. Data represent means ± SD (n = 3) and are expressed relative to the 37°C Control sample. **F)** Quantification for Fig. 3D of the protein levels of HSF1 against its loading control. Data represent means ± SD (n = 3) and are expressed relative to the 37°C empty control sample. **G)** Quantification for Fig. 3E of the protein levels of HSF1 against its loading control. Data represent means ± SD (n = 3) and are expressed relative to the HSF1-WT and empty vector samples. **H)** Comparison for the quantification of the protein levels of nuclear and cytoplasmic HSF1 before and after 5 minutes of heat shock. Data represent means ± SD (n = 3) and are expressed relative to the 37°C sample. **I)** MDA-MB-468 cells were incubated with either DMSO (0) or Bafilomycin A1 (BAF-A1). 16 hours later cells were lysed and nuclear and cytoplasmic fractions were analysed by western blot for the levels of the indicated proteins. **J)** MDA-MB-468 cells were incubated with either DMSO (0) or increasing concentrations of MG132 for the indicated times. Nuclear and cytoplasmic fractions were analysed by western blot for the levels of the indicated proteins. **K)** Quantification for Fig. 3F of the protein levels of HSF1 against its loading control. Data represent means ± SD (n = 3) and are expressed relative to the 37°C DMSO WT sample. **L)** Quantification for Fig. 3G of the protein levels of nuclear HSF1 against its loading control. Data represent means ± SD (n = 5) and are expressed relative to each own DMSO sample.

**Figure S4. Related to Figure 4. DYRK2 affects the expression levels of the HSF1 target gene *HSP70.* A)** Control (WT), DYRK2-KO or HSF1-KO MDA-MB-231 cells were incubated at 37 °C or 42 °C for one hour. The mRNA levels for *HSP70* (*HSPA1A*) were quantified using real-time PCR. The data were normalized using β-actin as an internal control. Data represent means ± SD (n=3) and are expressed relative to the control sample levels at 37 °C. *P ≤ 0.05, **P ≤ 0.01, ***P ≤ 0.001. **B)** Quantification for Fig. 4B of the protein levels of HSP70 against its loading control. Data represent means ± SD (n = 3) and are expressed relative to the 37°C WT sample. **C)** Control (WT) and DYRK2-KO MDA-MB-468 cells (*left panel*), or Control (WT) and DYRK2-KO MDA-MB-231 cells (*right panel*), were incubated with either vehicle or 20nM Bortezomib (Brtz) for four hours. The mRNA levels for *HSP70* (*HSPA1A*) were quantified using real-time PCR. The data were normalized using β-actin as an internal control. Data represent means ± SD (n=3) and are expressed relative to the control samples treated with vehicle. **P ≤ 0.01, ****P≤ 0.0001. **D)** Quantification for Fig. 4E of the protein levels of HSP70 against its loading control. Data represent means ± SD (n = 3) and are expressed relative to the 37°C control sample.

**Figure S5. Related to Figure 5. DYRK2 reduces sensitivity to proteotoxic stress via HSF1. A)** Quantification for Fig. 5B of the protein levels of PARP against its loading control. Data represent means ± SD (n = 3) and are expressed relative to the WT control (0) sample. **B)** Quantification for Fig. 5C of the protein levels of PARP against its loading control. Data represent means ± SD (n = 3) and are expressed relative to the control (0) sample. **C)** Quantification for Fig. 5D of the protein levels of PARP against its loading control. Data represent means ± SD (n = 3) and are expressed relative to the SiControl (0) sample **D)** Quantification for Fig. 5E of the protein levels of PARP against its loading control. Data represent means ± SD (n = 3) and are expressed relative to the SiControl (0) sample **E and F)** *Left panels*- Equal number of MDA-MB-468 WT or DYRK2-KO cells were seeded. After 24 hours, cells were exposed to increasing concentrations of doxorubicin (E) or paclitaxel (F). Three days later cell viability was measured using Alamar Blue assay. Data represent means ± SD (n=2 for doxorubicin and n=3 for paclitaxel). *Right panels-* WT and DYRK2-KO MDA-MB-468 cells were treated with either doxorubicin (E) or paclitaxel (F). 16 hours later, cells were lysed and the levels of apoptosis were analysed by western blotting using an antibody that recognises cleaved PARP. The corresponding quantifications of PARP protein levels against their loading controls are shown below. Data represent means ± SD (n=2).

**Figure S6. Related to Figure 6. DYRK2 levels correlate with HSF1 nuclear levels, prognosis and tumour recurrence in tissue from TNBC patients. A)** DYRK2 (*left panel*s) and HSF1 (*right panels*) antibody validation using Formalin-Fixed, Paraffin-Embedded (FFPE) cell pellets. **B)** Representative figures showing to show the different DYRK2 localisation observed and quantified: a) Negative both cytoplasmic and nuclear; b) Positive both cytoplasmic and nuclear; c) Positive cytoplasmic and negative nuclear; and d) Negative cytoplasmic and positive nuclear.  **C)** Representative samples showing high and low DYRK2 staining. **D)** Scatter plots showing correlation between cytoplasmic DYRK2 levels and cytoplasmic HSF1 levels (left panel) and between nuclear DYRK2 levels and nuclear HSF1 levels in every TNBC patient. Correlation coefficients (C.C) and p values (p) are indicated. **E)** *Upper panel-* DYRK2 mRNA expression in specific tumour samples (T) compared to equivalent normal tissues (N). Data was generated using GEPIA online platform and red indicates significant difference at p<0.05; Limma differential analysis Tumour vs Paired normal. *Lower panel-* Survival analysis carried out comparing DYRK2 high and low expressing patient tumour samples. GBM analysis carried out using the CGGA online platform and the other survival curves were derived utilising OncoLnc online platform with 24-33 percentile cut-offs. **F)** Tissue microarray details. **G)** Relationship between either cytoplasmic (left panel) or nuclear (right panel) DYRK2 levels and cancer-specific survival in patients with invasive ductal breast cancer (total cohort). **H)** Relationship between nuclear DYRK2 levels and overall survival in patients with triple negative (left panel) or triple negative and AR negative (right panel) invasive ductal breast cancer. **I)** Univariate and multivariate survival analysis for patients with triple negative and AR negative breast cancer to assess the relationship between clinico-pathological characteristics, DYRK2 and cancer specific survival.
