# Supplementary figures and images for: The stress-responsive kinase DYRK2 activates heat shock factor 1 promoting resistance to proteotoxic stress
Source: Cell Death Differ. 2020 Dec 2;28(5):1563–78. doi: 10.1038/s41418-020-00686-8 (PMC8166837; doi:10.1038/s41418-020-00686-8)

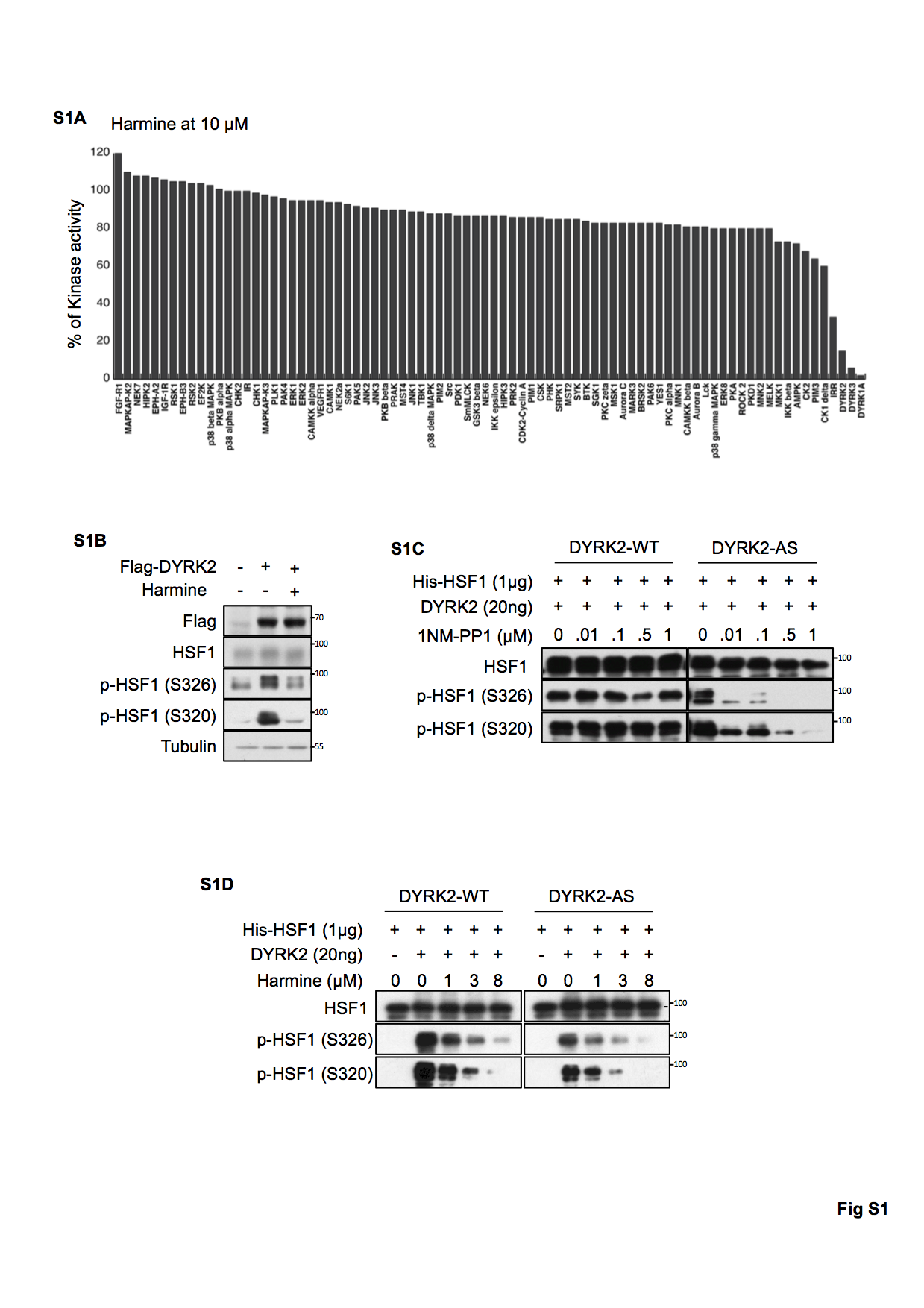

Supplement: Supplementary file 2 — Supplementary Figure 1 [file 41418_2020_686_MOESM2_ESM.tif]

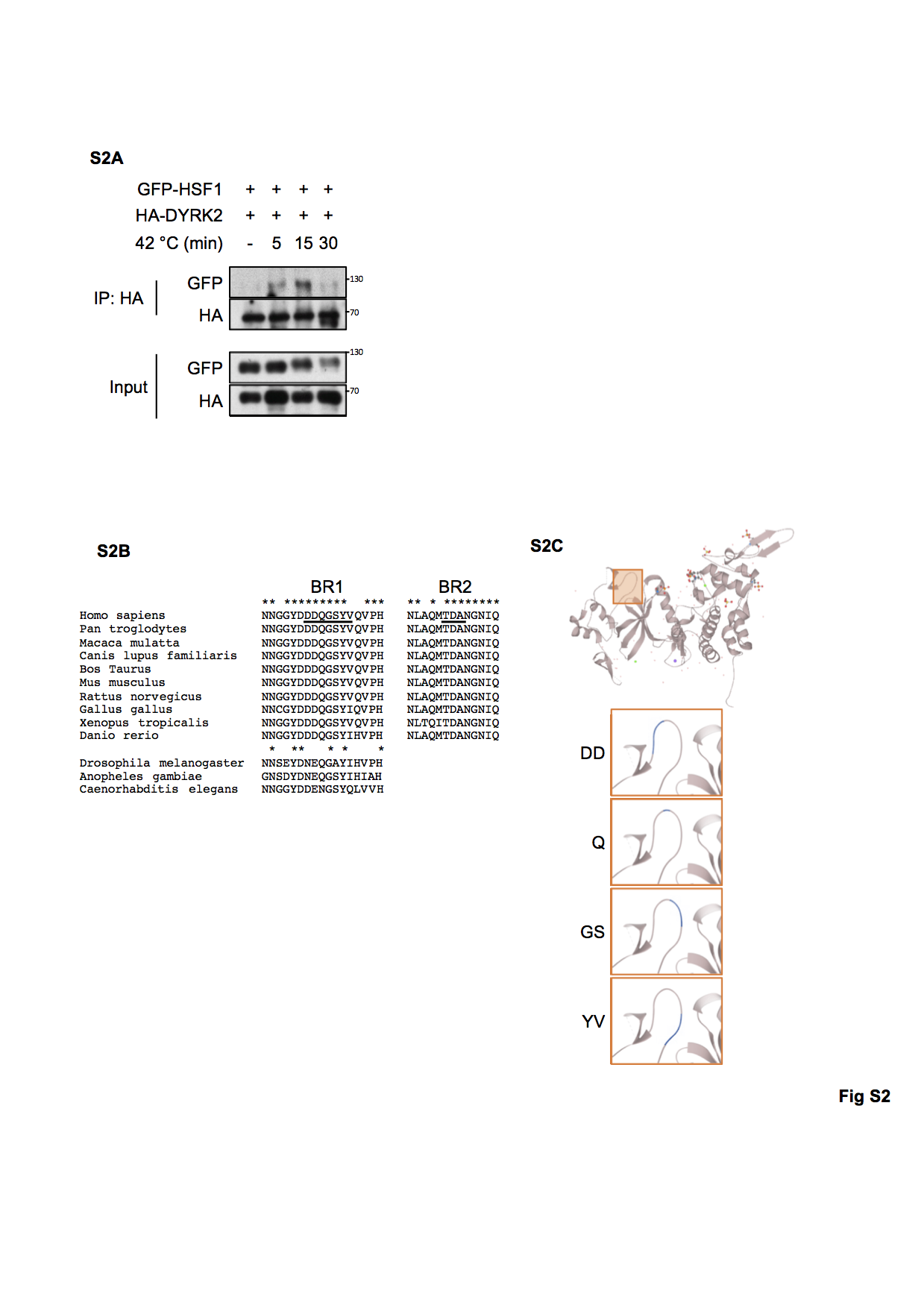

Supplement: Supplementary file 3 — Supplementary Figure 2 [file 41418_2020_686_MOESM3_ESM.tif]

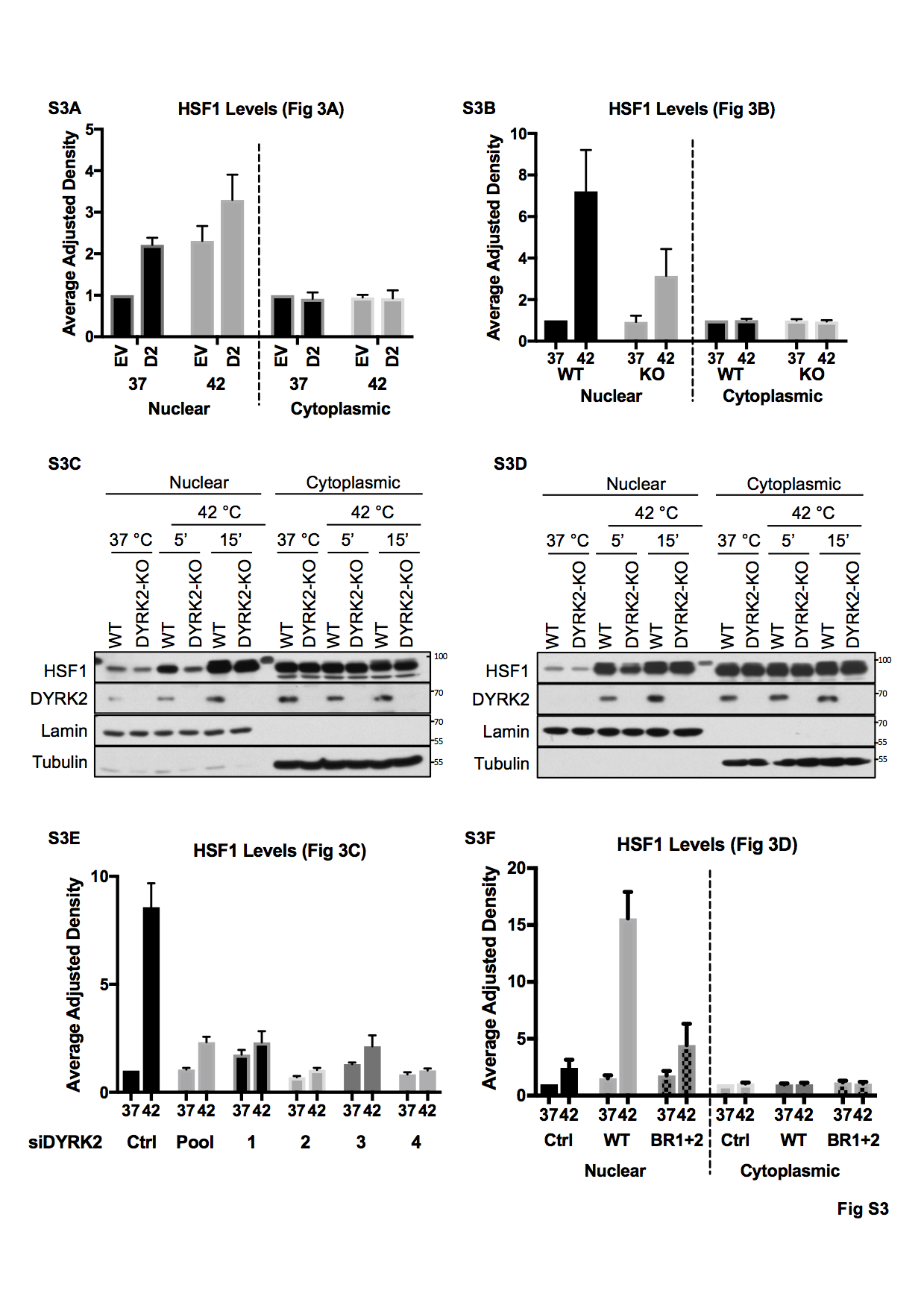

Supplement: Supplementary file 4 — Supplementary Figure 3 [file 41418_2020_686_MOESM4_ESM.tif]

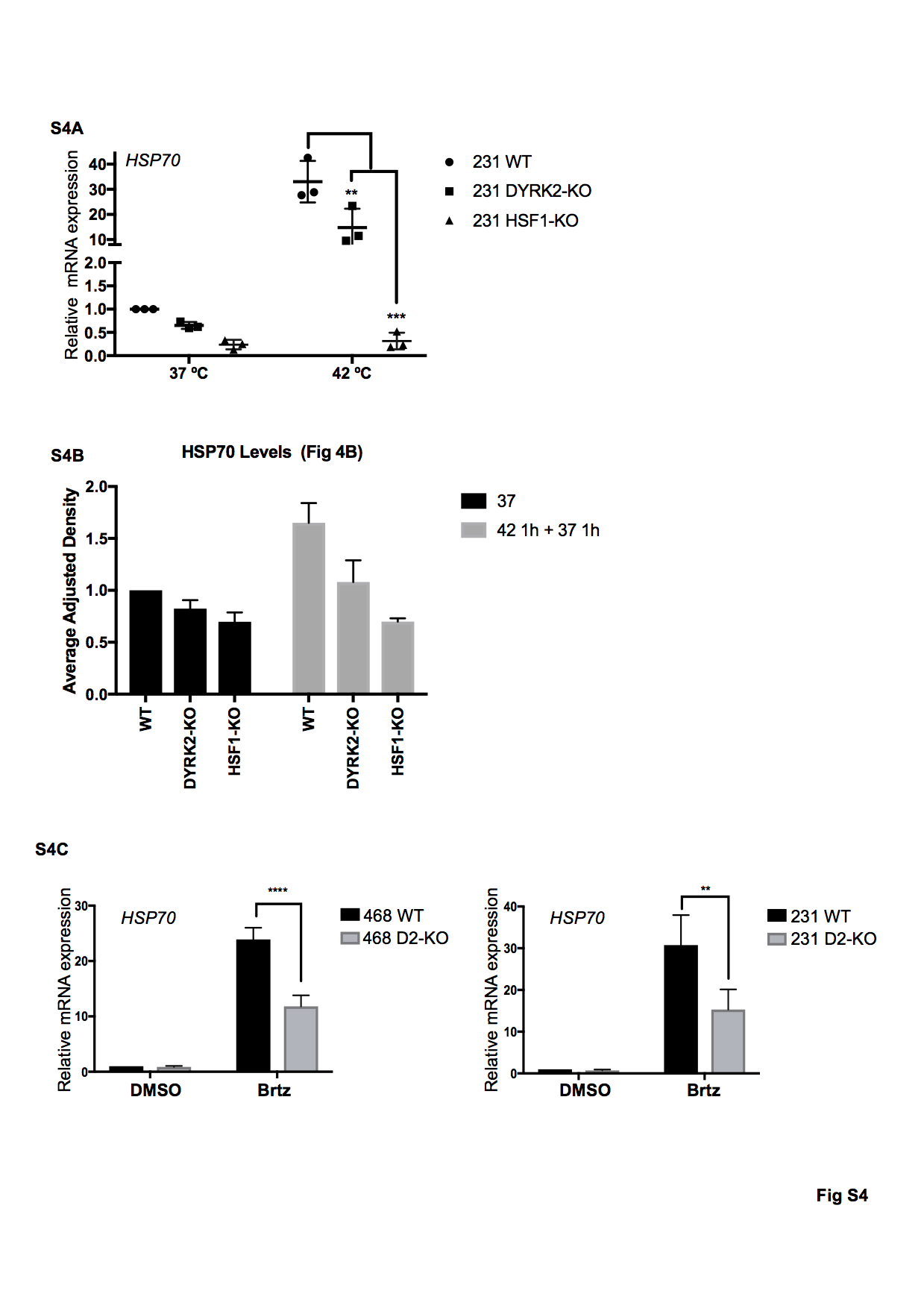

Supplement: Supplementary file 5 — Supplementary Figure 4 [file 41418_2020_686_MOESM5_ESM.tif]

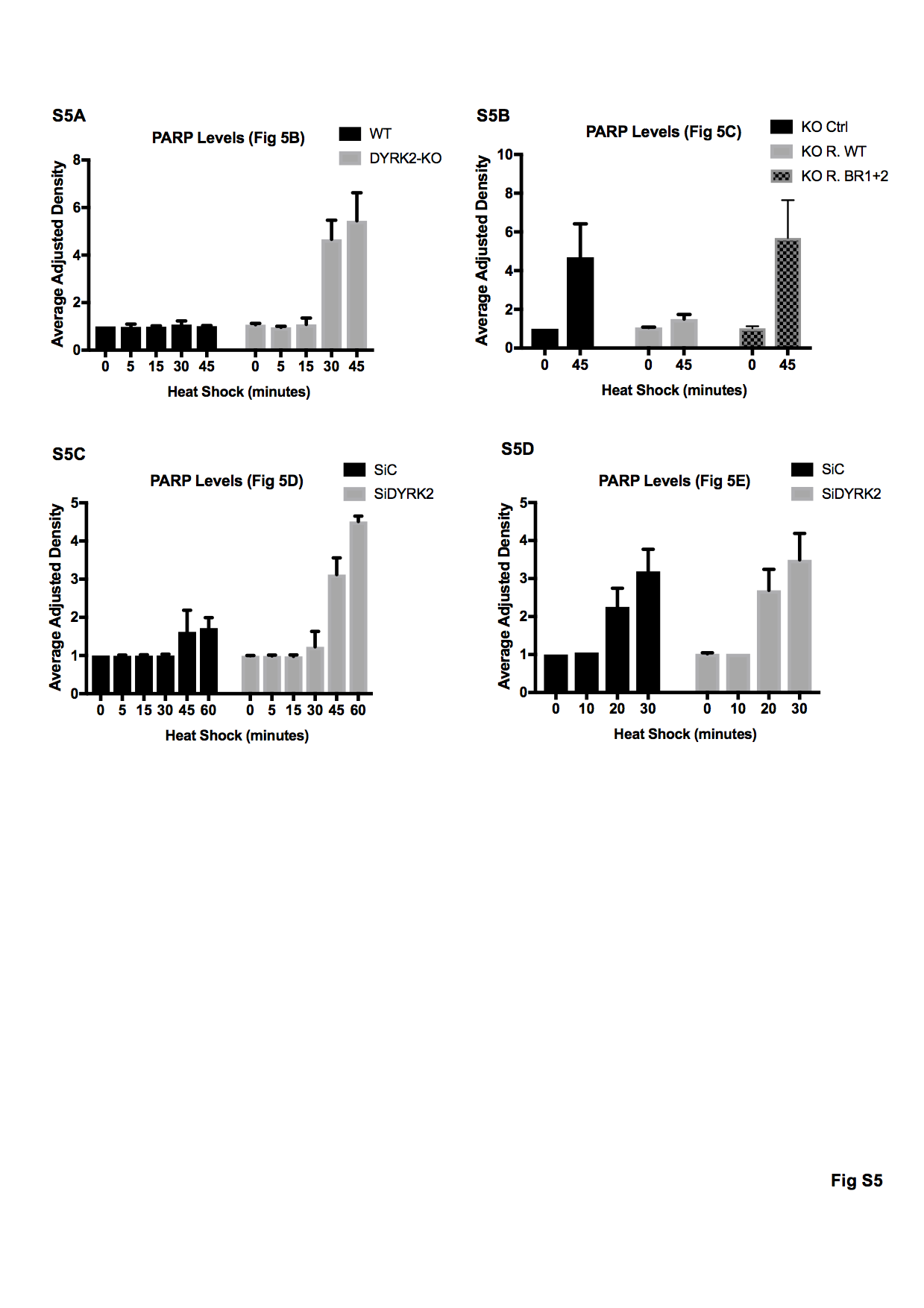

Supplement: Supplementary file 6 — Supplementary Figure 5 [file 41418_2020_686_MOESM6_ESM.tif]

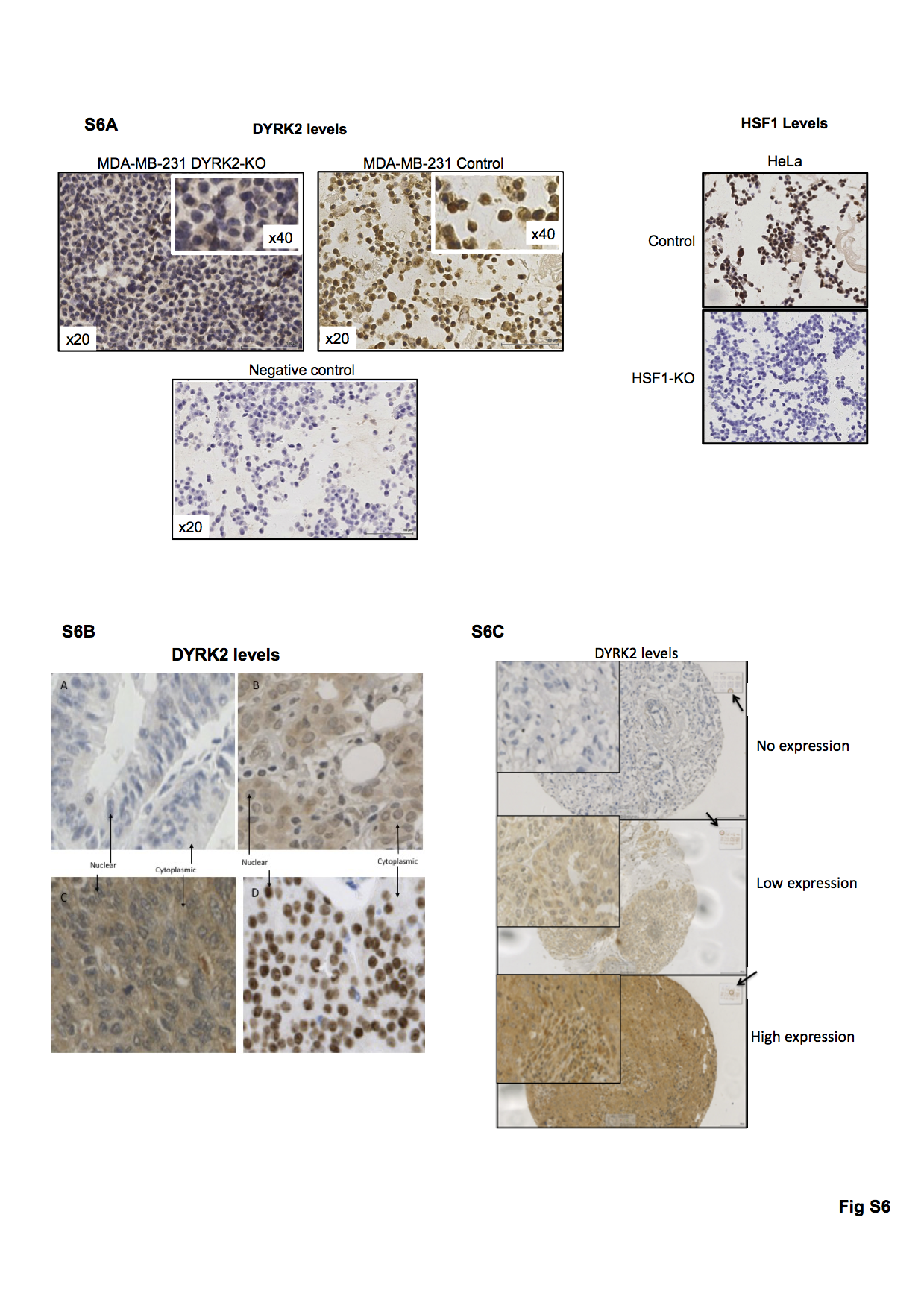

Supplement: Supplementary file 7 — Supplementary Figure 6 [file 41418_2020_686_MOESM7_ESM.tif]
